# 3-Hy­droxy-2-[(2*E*)-1-(2-hy­droxy-6-oxocyclo­hex-1-en-1-yl)-3-(2-meth­oxy­phen­yl)prop-2-en-1-yl]cyclo­hex-2-en-1-one

**DOI:** 10.1107/S1600536811038207

**Published:** 2011-09-30

**Authors:** Joo Hwan Cha, Myung Hee Son, Sun-Joon Min, Yong Seo Cho, Jae Kyun Lee

**Affiliations:** a Advanced Analysis Center, Korea Institute of Science & Technology, Hwarangro, 14-gil, Seongbuk-gu, Seoul, 136-791, South Korea; b Center for Neuro-Medicine, Korea Institute of Science & Technology, Hwarangro 14-gil, Seongbuk-gu, Seoul, 136-791, South Korea

## Abstract

In the title compound, C_22_H_24_O_5_, each of the cyclo­hexenone rings adopts a half-chair conformation. The hy­droxy and carbonyl O atoms face each other and are orientated to allow for the formation of the two intra­molecular O—H⋯O hydrogen bonds which are typical of xanthene derivatives. In the crystal, weak inter­molecular C—H⋯O hydrogen bonds link mol­ecules into layers parallel to the *ab* plane.

## Related literature

For the biological activity of xanthenes and their derivatives, see: Jonathan *et al.* (1988 ▶); Delfourne *et al.* (2000 ▶); Koeller *et al.* (2003 ▶); For related xanthene structures, see: Bolte *et al.* (2001 ▶); Palakshi Reddy *et al.* (2010 ▶).
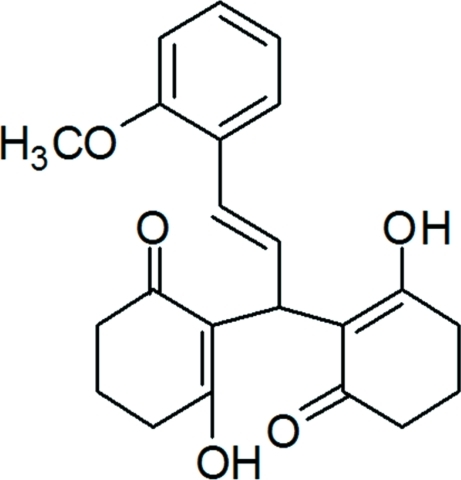


## Experimental

### 

#### Crystal data

C_22_H_24_O_5_
*M* *_r_* = 368.43
Monoclinic, 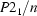

*a* = 10.7988 (8) Å
*b* = 12.0509 (8) Å
*c* = 15.0238 (10) Å
β = 104.536 (2)°
*V* = 1892.5 (3) Å^3^
*Z* = 4
Mo *K*α radiation
μ = 0.09 mm^−1^
*T* = 297 K
0.40 × 0.20 × 0.20 mm

#### Data collection

Rigaku R-AXIS RAPID diffractometer
Absorption correction: multi-scan (*ABSCOR*; Rigaku, 1995 ▶) *T*_min_ = 0.715, *T*_max_ = 0.982
18125 measured reflections
4304 independent reflections
2465 reflections with *F* ^2^ > 2.0σ(*F* ^2^)
*R*_int_ = 0.033

#### Refinement

*R*[*F* ^2^ > 2σ(*F* ^2^)] = 0.038
*wR*(*F* ^2^) = 0.119
*S* = 1.05
4304 reflections
255 parameters
H atoms treated by a mixture of independent and constrained refinement
Δρ_max_ = 0.18 e Å^−3^
Δρ_min_ = −0.20 e Å^−3^

### 

Data collection: *RAPID-AUTO* (Rigaku, 2006 ▶); cell refinement: *RAPID-AUTO*; data reduction: *RAPID-AUTO*; program(s) used to solve structure: *IL MILIONE* (Burla *et al.*, 2007 ▶); program(s) used to refine structure: *SHELXL97* (Sheldrick, 2008 ▶); molecular graphics: *CrystalStructure* (Rigaku, 2010 ▶); software used to prepare material for publication: *CrystalStructure*.

## Supplementary Material

Crystal structure: contains datablock(s) global, I. DOI: 10.1107/S1600536811038207/cv5143sup1.cif

Structure factors: contains datablock(s) I. DOI: 10.1107/S1600536811038207/cv5143Isup2.hkl

Supplementary material file. DOI: 10.1107/S1600536811038207/cv5143Isup3.cml

Additional supplementary materials:  crystallographic information; 3D view; checkCIF report

## Figures and Tables

**Table 1:** Hydrogen-bond geometry (Å, °)

*D*—H⋯*A*	*D*—H	H⋯*A*	*D*⋯*A*	*D*—H⋯*A*
O3—H3*A*⋯O5	0.82	1.85	2.644 (3)	163
O4—H4*A*⋯O2	0.82	1.80	2.594 (3)	162
C19—H19*A*⋯O4^i^	0.97	2.49	3.272 (3)	137

Symmetry code: (i) 
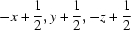
.

**Fig. 1.**
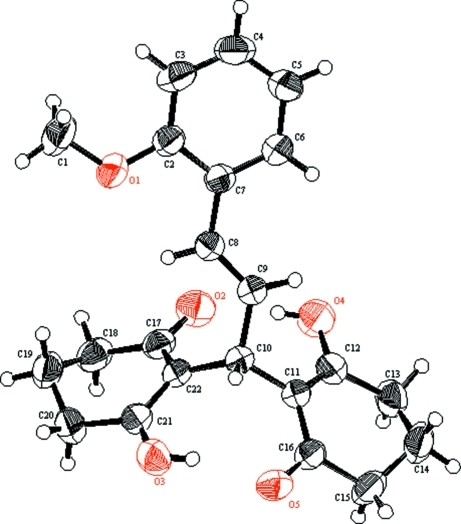
The molecular structure of (I) showing the atomic numbering and 50% probability displacement ellipsoids.

## supplementary crystallographic information

### Comment

Xanthenes constitute an important class of organic compounds that have attracted strong interest due to their useful biological and pharmacological properties, such as antibacterial, antiviral and antiinflammatory activities (Jonathan *et al.*, 1988). They also constitute a structural unit of a series of natural products (Delfourne *et al.*, 2000). Also they are being developed to act as new clinical agents in cancer therapy (Koeller *et al.*, 2003). Herewith we present the crystal structure of the title compound (I) (Fig. 1), which is a Xanthene derivative.

The molecular structure of xanthenedione features two cyclohexene rings, each has a half-chair conformation and lie above the respective least-squares plane through the remaining five carbon atoms (Palakshi Reddy *et al.*, 2010). Bolte and colleagues determined the crystal structures of bis-dimedone derivatives which showed nearly the same packing pattern irrespective of the different substituent in the *para* position of the aromatic ring (Bolte *et al.*, 2001). Two cyclohexenone rings in (I) display envelope conformation, and atoms C14 and C19 are directed towards the aromatic ring. The hydroxy and carbonyl O atoms face each other and are orientated to allow for the formation of two intramolecular O—H···O hydrogen bonds (Table 1) typical for Xanthene derivative.

In the crystal structure, weak intermolecular C—H···O hydrogen bonds (Table 1) links the molecules into layers parallel to *ab* plane.

### Experimental

To solution of 1,3-cyclohexanedione (4.68 mmol), 2-methoxycinnamaldehyde (1.87 mmol) and 4Å MS was added catalytic amounts of *L*-proline (0.47 mmol) in under nitrogen atmosphere. After stirring for 5 h, the anhydrous ethyl acetate (0.5 ml) was added to a reaction mixture and the solution was stirred for 2 days. The reaction mixture was filtered through pad of celite to remove MS and concentrated. The residue oil was purified by flash column chromatography to afford product which was recrystallized from ethanol to give crystals suitable for X-ray analysis.

### Refinement

Atoms H8 and H9 were located on a difference map and isotropically refined. All the rest H atoms were positioned geometrically and refined using a riding model with C—H = 0.93–0.98 Å and *U*_iso_(H) = 1.2 or 1.5 *U*_eq_(C). Rotating group model was applied for the methyl groups.

### Crystal data

**Table:**

C_22_H_24_O_5_	*F*(000) = 784.00
*M**_r_* = 368.43	*D*_x_ = 1.293 Mg m^−^^3^
Monoclinic, *P*2_1_/*n*	Mo *K*α radiation, λ = 0.71075 Å
Hall symbol: -P 2yn	Cell parameters from 11442 reflections
*a* = 10.7988 (8) Å	θ = 3.2–27.4°
*b* = 12.0509 (8) Å	µ = 0.09 mm^−^^1^
*c* = 15.0238 (10) Å	*T* = 297 K
β = 104.536 (2)°	Chunk, colourless
*V* = 1892.5 (3) Å^3^	0.40 × 0.20 × 0.20 mm
*Z* = 4	

### Data collection

**Table:**

Rigaku R-AXIS RAPID diffractometer	2465 reflections with *F*^2^ > 2.0σ(*F*^2^)
Detector resolution: 10.000 pixels mm^-1^	*R*_int_ = 0.033
ω scans	θ_max_ = 27.5°
Absorption correction: multi-scan (*ABSCOR*; Rigaku, 1995)	*h* = −14→13
*T*_min_ = 0.715, *T*_max_ = 0.982	*k* = −15→13
18125 measured reflections	*l* = −19→19
4304 independent reflections	

### Refinement

**Table:**

Refinement on *F*^2^	Secondary atom site location: difference Fourier map
*R*[*F*^2^ > 2σ(*F*^2^)] = 0.038	Hydrogen site location: inferred from neighbouring sites
*wR*(*F*^2^) = 0.119	H atoms treated by a mixture of independent and constrained refinement
*S* = 1.05	*w* = 1/[σ^2^(*F*_o_^2^) + (0.0612*P*)^2^ + 0.0196*P*] where *P* = (*F*_o_^2^ + 2*F*_c_^2^)/3
4304 reflections	(Δ/σ)_max_ < 0.001
255 parameters	Δρ_max_ = 0.18 e Å^−^^3^
0 restraints	Δρ_min_ = −0.20 e Å^−^^3^
Primary atom site location: structure-invariant direct methods	

### Special details

**Table:**

Geometry. All e.s.d.'s (except the e.s.d. in the dihedral angle between two l.s. planes) are estimated using the full covariance matrix. The cell e.s.d.'s are taken into account individually in the estimation of e.s.d.'s in distances, angles and torsion angles; correlations between e.s.d.'s in cell parameters are only used when they are defined by crystal symmetry. An approximate (isotropic) treatment of cell e.s.d.'s is used for estimating e.s.d.'s involving l.s. planes.
Refinement. Refinement was performed using all reflections. The weighted *R*-factor (*wR*) and goodness of fit (*S*) are based on *F*^2^. *R*-factor (gt) are based on *F*. The threshold expression of *F*^2^ > 2.0 σ(*F*^2^) is used only for calculating *R*-factor (gt).

### Fractional atomic coordinates and isotropic or equivalent isotropic displacement parameters (Å^2^)

**Table:**

	*x*	*y*	*z*	*U*_iso_*/*U*_eq_	
O1	0.09043 (11)	0.05978 (9)	−0.07867 (8)	0.0658 (4)	
O2	0.28313 (11)	0.19541 (10)	0.23571 (8)	0.0645 (4)	
O3	0.41828 (12)	0.47367 (10)	0.06364 (9)	0.0663 (4)	
O4	0.51114 (12)	0.11253 (10)	0.29205 (9)	0.0724 (4)	
O5	0.65368 (11)	0.41783 (9)	0.15245 (8)	0.0632 (4)	
C1	−0.02975 (16)	0.04933 (18)	−0.14336 (13)	0.0742 (6)	
C2	0.15960 (14)	−0.03397 (12)	−0.04910 (10)	0.0488 (4)	
C3	0.11998 (17)	−0.13977 (14)	−0.07901 (12)	0.0626 (5)	
C4	0.19488 (18)	−0.23024 (14)	−0.04442 (14)	0.0678 (5)	
C5	0.30822 (16)	−0.21703 (13)	0.02058 (13)	0.0625 (5)	
C6	0.34792 (15)	−0.11098 (12)	0.05032 (11)	0.0520 (4)	
C7	0.27626 (13)	−0.01761 (12)	0.01666 (10)	0.0434 (4)	
C8	0.31364 (14)	0.09541 (12)	0.05010 (10)	0.0446 (4)	
C9	0.42886 (14)	0.12987 (12)	0.09303 (10)	0.0459 (4)	
C10	0.46403 (12)	0.24820 (11)	0.12552 (10)	0.0416 (4)	
C11	0.58135 (13)	0.25288 (11)	0.20732 (10)	0.0429 (4)	
C12	0.59878 (15)	0.18020 (13)	0.28119 (11)	0.0517 (4)	
C13	0.72021 (16)	0.17659 (15)	0.35550 (12)	0.0646 (5)	
C14	0.83130 (17)	0.22703 (16)	0.32580 (14)	0.0732 (6)	
C15	0.79534 (15)	0.34015 (16)	0.28366 (13)	0.0653 (5)	
C16	0.67165 (14)	0.33779 (13)	0.21009 (11)	0.0497 (4)	
C17	0.26577 (14)	0.28332 (13)	0.18620 (11)	0.0492 (4)	
C18	0.14924 (15)	0.35094 (15)	0.18728 (12)	0.0622 (5)	
C19	0.10349 (16)	0.41715 (15)	0.10015 (13)	0.0679 (6)	
C20	0.21250 (16)	0.48847 (15)	0.08684 (13)	0.0664 (5)	
C21	0.33291 (15)	0.42426 (12)	0.09539 (11)	0.0492 (4)	
C22	0.35061 (13)	0.31784 (11)	0.13452 (10)	0.0417 (4)	
H1A	−0.0834	−0.0000	−0.1195	0.0890*	
H1B	−0.0175	0.0201	−0.1999	0.0890*	
H1C	−0.0698	0.1209	−0.1544	0.0890*	
H3	0.0427	−0.1499	−0.1225	0.0751*	
H3A	0.4882	0.4448	0.0851	0.0795*	
H4	0.1682	−0.3010	−0.0655	0.0813*	
H4A	0.4409	0.1351	0.2632	0.0868*	
H5	0.3578	−0.2783	0.0444	0.0750*	
H6	0.4251	−0.1021	0.0942	0.0624*	
H8	0.2464 (14)	0.1475 (12)	0.0421 (10)	0.043 (4)*	
H9	0.4989 (16)	0.0791 (13)	0.1032 (11)	0.057 (5)*	
H10	0.4924	0.2827	0.0750	0.0499*	
H13A	0.7402	0.1001	0.3735	0.0776*	
H13B	0.7081	0.2163	0.4088	0.0776*	
H14A	0.8555	0.1789	0.2812	0.0878*	
H14B	0.9042	0.2339	0.3785	0.0878*	
H15A	0.7873	0.3917	0.3315	0.0783*	
H15B	0.8631	0.3668	0.2573	0.0783*	
H18A	0.1692	0.4011	0.2394	0.0747*	
H18B	0.0813	0.3019	0.1947	0.0747*	
H19A	0.0318	0.4636	0.1044	0.0815*	
H19B	0.0753	0.3675	0.0482	0.0815*	
H20A	0.1881	0.5225	0.0265	0.0797*	
H20B	0.2280	0.5474	0.1322	0.0797*	

### Atomic displacement parameters (Å^2^)

**Table:**

	*U*^11^	*U*^22^	*U*^33^	*U*^12^	*U*^13^	*U*^23^
O1	0.0519 (7)	0.0618 (7)	0.0705 (8)	−0.0035 (6)	−0.0095 (6)	0.0080 (6)
O2	0.0689 (8)	0.0638 (8)	0.0656 (8)	−0.0072 (6)	0.0257 (6)	0.0071 (6)
O3	0.0630 (8)	0.0536 (7)	0.0817 (9)	0.0034 (6)	0.0172 (7)	0.0186 (6)
O4	0.0691 (8)	0.0665 (8)	0.0769 (9)	−0.0077 (7)	0.0098 (7)	0.0239 (7)
O5	0.0572 (7)	0.0508 (7)	0.0782 (8)	−0.0137 (5)	0.0107 (6)	0.0069 (6)
C1	0.0495 (10)	0.0975 (15)	0.0642 (11)	−0.0084 (10)	−0.0070 (9)	0.0180 (10)
C2	0.0461 (9)	0.0512 (9)	0.0466 (9)	−0.0057 (7)	0.0067 (7)	0.0001 (7)
C3	0.0601 (10)	0.0625 (11)	0.0596 (10)	−0.0195 (9)	0.0046 (8)	−0.0101 (9)
C4	0.0716 (12)	0.0504 (10)	0.0816 (13)	−0.0154 (9)	0.0199 (10)	−0.0154 (9)
C5	0.0592 (11)	0.0444 (9)	0.0842 (13)	−0.0005 (8)	0.0184 (10)	−0.0037 (9)
C6	0.0430 (9)	0.0493 (9)	0.0618 (10)	−0.0009 (7)	0.0095 (8)	−0.0019 (8)
C7	0.0418 (8)	0.0425 (8)	0.0454 (8)	−0.0040 (6)	0.0096 (7)	−0.0015 (7)
C8	0.0433 (8)	0.0420 (8)	0.0456 (8)	0.0013 (7)	0.0058 (7)	−0.0019 (7)
C9	0.0421 (8)	0.0419 (8)	0.0511 (9)	−0.0004 (7)	0.0073 (7)	−0.0022 (7)
C10	0.0390 (8)	0.0400 (7)	0.0442 (8)	−0.0033 (6)	0.0076 (7)	−0.0007 (7)
C11	0.0386 (8)	0.0391 (7)	0.0492 (8)	−0.0009 (6)	0.0077 (7)	−0.0026 (7)
C12	0.0526 (9)	0.0446 (8)	0.0553 (9)	0.0035 (7)	0.0084 (8)	0.0009 (7)
C13	0.0668 (12)	0.0593 (10)	0.0562 (10)	0.0119 (9)	−0.0062 (9)	0.0020 (9)
C14	0.0473 (10)	0.0858 (13)	0.0741 (12)	0.0122 (9)	−0.0079 (9)	−0.0055 (11)
C15	0.0421 (9)	0.0754 (11)	0.0720 (11)	−0.0108 (8)	0.0026 (8)	−0.0105 (10)
C16	0.0414 (8)	0.0488 (8)	0.0568 (9)	−0.0024 (7)	0.0086 (7)	−0.0072 (8)
C17	0.0448 (9)	0.0504 (9)	0.0507 (9)	−0.0078 (7)	0.0086 (7)	−0.0110 (8)
C18	0.0466 (9)	0.0694 (11)	0.0731 (12)	−0.0054 (8)	0.0193 (8)	−0.0233 (10)
C19	0.0439 (9)	0.0700 (11)	0.0824 (13)	0.0101 (8)	0.0016 (9)	−0.0209 (10)
C20	0.0605 (11)	0.0552 (10)	0.0769 (12)	0.0145 (8)	0.0047 (9)	−0.0055 (9)
C21	0.0475 (9)	0.0467 (8)	0.0486 (9)	−0.0012 (7)	0.0031 (7)	−0.0041 (7)
C22	0.0377 (8)	0.0413 (8)	0.0427 (8)	−0.0030 (6)	0.0034 (6)	−0.0046 (7)

### Geometric parameters (Å, °)

**Table:**

O1—C1	1.4183 (19)	C20—C21	1.491 (3)
O1—C2	1.3657 (18)	C21—C22	1.404 (2)
O2—C17	1.281 (2)	O3—H3A	0.820
O3—C21	1.286 (3)	O4—H4A	0.820
O4—C12	1.290 (3)	C1—H1A	0.960
O5—C16	1.278 (2)	C1—H1B	0.960
C2—C3	1.383 (3)	C1—H1C	0.960
C2—C7	1.4061 (19)	C3—H3	0.930
C3—C4	1.379 (3)	C4—H4	0.930
C4—C5	1.370 (3)	C5—H5	0.930
C5—C6	1.386 (3)	C6—H6	0.930
C6—C7	1.387 (2)	C8—H8	0.944 (15)
C7—C8	1.473 (2)	C9—H9	0.954 (17)
C8—C9	1.317 (2)	C10—H10	0.980
C9—C10	1.524 (2)	C13—H13A	0.970
C10—C11	1.5287 (18)	C13—H13B	0.970
C10—C22	1.519 (2)	C14—H14A	0.970
C11—C12	1.389 (3)	C14—H14B	0.970
C11—C16	1.407 (2)	C15—H15A	0.970
C12—C13	1.494 (2)	C15—H15B	0.970
C13—C14	1.509 (3)	C18—H18A	0.970
C14—C15	1.512 (3)	C18—H18B	0.970
C15—C16	1.505 (2)	C19—H19A	0.970
C17—C18	1.503 (3)	C19—H19B	0.970
C17—C22	1.404 (3)	C20—H20A	0.970
C18—C19	1.507 (3)	C20—H20B	0.970
C19—C20	1.511 (3)		
			
O1···C8	2.7196 (17)	C8···H13B^i^	3.1204
O2···O4	2.5943 (17)	C8···H15A^i^	3.2285
O2···C8	3.130 (2)	C9···H6^iv^	3.5698
O2···C9	3.064 (3)	C9···H14B^i^	3.5664
O2···C10	2.930 (2)	C10···H5^iv^	3.5801
O2···C11	3.424 (2)	C11···H1B^viii^	3.3634
O2···C12	3.309 (2)	C11···H1C^viii^	3.3161
O2···C21	3.593 (2)	C12···H1C^viii^	3.2985
O3···O5	2.6443 (16)	C13···H8^viii^	3.470 (15)
O3···C10	2.8741 (18)	C13···H20A^viii^	3.5946
O3···C11	3.5961 (18)	C14···H5^v^	3.4339
O3···C16	3.4622 (19)	C14···H6^v^	3.3192
O3···C17	3.590 (3)	C15···H4^iv^	3.4312
O4···C9	2.905 (2)	C15···H6^v^	3.1913
O4···C10	2.924 (2)	C16···H1B^viii^	3.2120
O4···C17	3.4162 (19)	C16···H4^iv^	3.1300
O4···C22	3.5593 (18)	C17···H1B^viii^	3.4654
O5···C10	2.8497 (18)	C17···H1C^viii^	2.8440
O5···C12	3.587 (2)	C18···H1C^viii^	3.3703
O5···C21	3.354 (2)	C18···H3^vi^	3.1780
O5···C22	3.4341 (19)	C18···H4^vi^	3.5086
C1···C3	2.821 (3)	C18···H15B^xii^	3.5102
C2···C5	2.774 (3)	C19···H4^vi^	3.1730
C3···C6	2.748 (3)	C19···H4A^ix^	3.4414
C4···C7	2.788 (3)	C19···H19A^xiii^	3.3752
C6···C9	3.053 (2)	C19···H20A^xiii^	3.3249
C8···C17	3.179 (3)	C20···H4^x^	3.3690
C8···C22	2.949 (2)	C20···H4A^ix^	3.5808
C9···C12	3.020 (2)	C20···H5^x^	3.3566
C9···C17	3.117 (3)	C20···H13A^i^	3.4621
C11···C14	2.853 (2)	C20···H19A^xiii^	3.4272
C11···C17	3.362 (2)	C21···H1B^viii^	3.1656
C11···C21	3.468 (2)	C21···H13A^i^	3.2451
C12···C15	2.861 (3)	C21···H13B^i^	3.2631
C12···C22	3.439 (2)	C22···H1B^viii^	3.2018
C13···C16	2.872 (3)	C22···H1C^viii^	3.1582
C16···C22	3.375 (2)	C22···H13B^i^	3.3789
C17···C20	2.871 (3)	H1A···O1^vi^	3.0828
C18···C21	2.828 (3)	H1A···O2^vi^	3.3695
C19···C22	2.851 (3)	H1A···C2^vi^	2.8827
O1···C15^i^	3.595 (3)	H1A···C3^vi^	3.5331
O3···O3^ii^	2.978 (2)	H1A···C6^vi^	3.5387
O3···O5^ii^	3.4039 (18)	H1A···C7^vi^	2.8961
O4···C19^iii^	3.272 (3)	H1A···C8^vi^	3.1454
O5···O3^ii^	3.4039 (18)	H1A···H1A^vi^	3.5908
O5···C4^iv^	3.428 (3)	H1A···H8^vi^	2.9387
O5···C13^v^	3.417 (3)	H1A···H18A^i^	3.1954
C1···C2^vi^	3.521 (3)	H1B···O3^i^	3.4446
C1···C17^i^	3.558 (3)	H1B···O5^i^	3.3091
C2···C1^vi^	3.521 (3)	H1B···C11^i^	3.3634
C4···O5^iv^	3.428 (3)	H1B···C16^i^	3.2120
C4···C16^iv^	3.428 (3)	H1B···C17^i^	3.4654
C13···O5^vii^	3.417 (3)	H1B···C21^i^	3.1656
C15···O1^viii^	3.595 (3)	H1B···C22^i^	3.2018
C16···C4^iv^	3.428 (3)	H1B···H3A^i^	3.2729
C17···C1^viii^	3.558 (3)	H1B···H14A^iv^	3.3723
C19···O4^ix^	3.272 (3)	H1B···H15A^i^	3.3772
O1···H3	2.6295	H1B···H18A^i^	3.4114
O1···H8	2.390 (14)	H1B···H20B^i^	3.3308
O2···H4A	1.8023	H1C···O2^i^	2.9723
O2···H8	2.893 (15)	H1C···O4^i^	3.4764
O2···H18A	2.7740	H1C···C2^vi^	3.5878
O2···H18B	2.4700	H1C···C11^i^	3.3161
O3···H10	2.4282	H1C···C12^i^	3.2985
O3···H20A	2.4779	H1C···C17^i^	2.8440
O3···H20B	2.6713	H1C···C18^i^	3.3703
O4···H9	2.835 (17)	H1C···C22^i^	3.1582
O4···H13A	2.4733	H1C···H4A^i^	3.2039
O4···H13B	2.7005	H1C···H13B^i^	3.5103
O5···H3A	1.8483	H1C···H18A^i^	2.8820
O5···H10	2.4540	H3···O2^vi^	3.5452
O5···H15A	2.7329	H3···C18^vi^	3.1780
O5···H15B	2.4876	H3···H14A^iv^	2.8866
C1···H3	2.5202	H3···H18B^vi^	2.3650
C2···H1A	2.6034	H3···H19B^vi^	3.2350
C2···H1B	2.6535	H3A···O3^ii^	2.8477
C2···H1C	3.1862	H3A···H1B^viii^	3.2729
C2···H4	3.2307	H3A···H3A^ii^	2.9488
C2···H6	3.2298	H3A···H5^iv^	3.4928
C2···H8	2.626 (15)	H3A···H13A^v^	3.4020
C3···H1A	2.7126	H4···O5^iv^	2.9416
C3···H1B	2.8037	H4···C15^iv^	3.4312
C3···H5	3.2300	H4···C16^iv^	3.1300
C4···H6	3.2099	H4···C18^vi^	3.5086
C5···H3	3.2255	H4···C19^vi^	3.1730
C6···H4	3.2162	H4···C20^xi^	3.3690
C6···H8	3.294 (15)	H4···H14A^iv^	3.5105
C6···H9	2.809 (16)	H4···H15B^iv^	2.9262
C7···H3	3.2601	H4···H18B^vi^	2.8985
C7···H5	3.2616	H4···H19A^vi^	2.8654
C7···H9	2.694 (16)	H4···H19B^vi^	2.8224
C8···H4A	3.1867	H4···H20A^xi^	2.5151
C8···H6	2.6746	H4···H20B^xi^	3.4074
C8···H10	2.9324	H4A···C19^iii^	3.4414
C9···H4A	2.5271	H4A···C20^iii^	3.5808
C9···H6	2.7961	H4A···H1C^viii^	3.2039
C10···H3A	2.4763	H4A···H18A^iii^	3.0575
C10···H4A	2.5403	H4A···H19A^iii^	2.8330
C10···H8	2.662 (14)	H4A···H20B^iii^	2.8906
C11···H3A	2.9679	H5···O3^xi^	3.0571
C11···H4A	2.3780	H5···O5^iv^	3.3769
C11···H9	2.631 (16)	H5···C10^iv^	3.5801
C11···H13A	3.2271	H5···C14^vii^	3.4339
C11···H13B	3.0252	H5···C20^xi^	3.3566
C11···H14A	3.0235	H5···H3A^iv^	3.4928
C11···H15A	3.0236	H5···H10^iv^	2.7005
C11···H15B	3.2498	H5···H14A^vii^	3.5555
C12···H9	2.889 (16)	H5···H14B^vii^	2.5446
C12···H10	3.2647	H5···H20A^xi^	2.9903
C12···H14A	2.7721	H5···H20B^xi^	3.0067
C12···H14B	3.3185	H6···C9^iv^	3.5698
C12···H15A	3.2309	H6···C14^vii^	3.3192
C13···H4A	3.0282	H6···C15^vii^	3.1913
C13···H15A	2.7395	H6···H9^iv^	3.2824
C13···H15B	3.3097	H6···H14B^vii^	2.6633
C15···H13A	3.3091	H6···H15A^vii^	3.0262
C15···H13B	2.7458	H6···H15B^vii^	2.7899
C16···H3A	2.6923	H6···H18A^iii^	2.9252
C16···H10	2.5172	H6···H18B^iii^	3.3928
C16···H13B	3.2591	H8···C13^i^	3.470 (15)
C16···H14A	2.7721	H8···H1A^vi^	2.9387
C16···H14B	3.3296	H8···H13B^i^	2.5419
C17···H4A	2.6467	H8···H15A^i^	3.3368
C17···H8	2.680 (15)	H9···C5^iv^	3.536 (19)
C17···H10	3.2916	H9···C6^iv^	3.181 (19)
C17···H19A	3.3245	H9···C7^iv^	3.448 (19)
C17···H19B	2.7239	H9···H6^iv^	3.2824
C17···H20B	3.2846	H9···H15A^vii^	3.2033
C18···H20A	3.2862	H9···H15B^vii^	3.4048
C18···H20B	2.7145	H10···C4^iv^	3.5768
C20···H3A	3.0298	H10···C5^iv^	2.9791
C20···H18A	2.6689	H10···C6^iv^	3.5256
C20···H18B	3.2925	H10···H5^iv^	2.7005
C21···H8	3.502 (15)	H10···H13B^i^	3.4366
C21···H10	2.4969	H10···H14B^i^	2.8717
C21···H18A	3.1320	H13A···O3^viii^	3.1455
C21···H19A	3.3218	H13A···O5^vii^	2.5517
C21···H19B	2.7781	H13A···C20^viii^	3.4621
C22···H3A	2.3781	H13A···C21^viii^	3.2451
C22···H4A	2.9304	H13A···H3A^vii^	3.4020
C22···H8	2.574 (14)	H13A···H15B^vii^	3.4506
C22···H9	3.382 (17)	H13A···H19A^iii^	3.4546
C22···H18A	2.9800	H13A···H19B^viii^	3.5436
C22···H18B	3.2603	H13A···H20A^viii^	2.9036
C22···H19B	2.9905	H13B···O1^viii^	3.0081
C22···H20A	3.2202	H13B···C8^viii^	3.1204
C22···H20B	3.0637	H13B···C21^viii^	3.2631
H1A···H3	2.2690	H13B···C22^viii^	3.3789
H1B···H3	2.3646	H13B···H1C^viii^	3.5103
H1C···H3	3.4728	H13B···H8^viii^	2.5419
H3···H4	2.3031	H13B···H10^viii^	3.4366
H3A···H10	1.9610	H13B···H19B^viii^	2.9943
H3A···H20A	3.2743	H13B···H20A^viii^	3.4126
H3A···H20B	3.3049	H14A···O5^vii^	3.3092
H4···H5	2.3014	H14A···C3^iv^	3.1507
H4A···H8	3.4579	H14A···C4^iv^	3.5164
H4A···H9	2.7158	H14A···H1B^iv^	3.3723
H4A···H10	3.5005	H14A···H3^iv^	2.8866
H4A···H13A	3.2681	H14A···H4^iv^	3.5105
H4A···H13B	3.3016	H14A···H5^v^	3.5555
H5···H6	2.3077	H14A···H18B^xiv^	3.3802
H6···H8	3.5527	H14B···C5^v^	3.1512
H6···H9	2.3176	H14B···C6^v^	3.2197
H8···H9	2.77 (3)	H14B···C9^viii^	3.5664
H8···H10	3.0491	H14B···H5^v^	2.5446
H8···H19B	3.2466	H14B···H6^v^	2.6633
H9···H10	2.4876	H14B···H10^viii^	2.8717
H13A···H14A	2.2890	H15A···O1^viii^	2.8504
H13A···H14B	2.3826	H15A···C2^viii^	3.0500
H13B···H14A	2.8184	H15A···C7^viii^	3.1974
H13B···H14B	2.2847	H15A···C8^viii^	3.2285
H13B···H15A	2.6544	H15A···H1B^viii^	3.3772
H14A···H15A	2.8224	H15A···H6^v^	3.0262
H14A···H15B	2.2972	H15A···H8^viii^	3.3368
H14B···H15A	2.2932	H15A···H9^v^	3.2033
H14B···H15B	2.3813	H15B···O4^v^	3.4160
H18A···H19A	2.3178	H15B···C4^iv^	3.5109
H18A···H19B	2.8261	H15B···C18^xiv^	3.5102
H18A···H20A	3.5710	H15B···H4^iv^	2.9262
H18A···H20B	2.5737	H15B···H6^v^	2.7899
H18B···H19A	2.3578	H15B···H9^v^	3.4048
H18B···H19B	2.3246	H15B···H13A^v^	3.4506
H18B···H20B	3.5909	H15B···H18A^xiv^	3.4073
H19A···H20A	2.3907	H15B···H18B^xiv^	2.8571
H19A···H20B	2.2891	H15B···H19A^xiv^	3.4726
H19B···H20A	2.2973	H18A···O2^ix^	3.5903
H19B···H20B	2.8231	H18A···O4^ix^	3.1696
O1···H1A^vi^	3.0828	H18A···C1^viii^	3.3400
O1···H13B^i^	3.0081	H18A···C6^ix^	3.2131
O1···H15A^i^	2.8504	H18A···H1A^viii^	3.1954
O2···H1A^vi^	3.3695	H18A···H1B^viii^	3.4114
O2···H1C^viii^	2.9723	H18A···H1C^viii^	2.8820
O2···H3^vi^	3.5452	H18A···H4A^ix^	3.0575
O2···H18A^iii^	3.5903	H18A···H6^ix^	2.9252
O2···H20B^iii^	2.6927	H18A···H15B^xii^	3.4073
O3···H1B^viii^	3.4446	H18B···C3^vi^	3.1087
O3···H3A^ii^	2.8477	H18B···C4^vi^	3.3700
O3···H5^x^	3.0571	H18B···H3^vi^	2.3650
O3···H13A^i^	3.1455	H18B···H4^vi^	2.8985
O4···H1C^viii^	3.4764	H18B···H6^ix^	3.3928
O4···H15B^vii^	3.4160	H18B···H14A^xii^	3.3802
O4···H18A^iii^	3.1696	H18B···H15B^xii^	2.8571
O4···H19A^iii^	2.4938	H19A···O4^ix^	2.4938
O4···H20B^iii^	3.1709	H19A···C19^xiii^	3.3752
O5···H1B^viii^	3.3091	H19A···C20^xiii^	3.4272
O5···H4^iv^	2.9416	H19A···H4^vi^	2.8654
O5···H5^iv^	3.3769	H19A···H4A^ix^	2.8330
O5···H13A^v^	2.5517	H19A···H13A^ix^	3.4546
O5···H14A^v^	3.3092	H19A···H15B^xii^	3.4726
C1···H18A^i^	3.3400	H19A···H19A^xiii^	3.1627
C2···H1A^vi^	2.8827	H19A···H19B^xiii^	3.0665
C2···H1C^vi^	3.5878	H19A···H20A^xiii^	2.6815
C2···H15A^i^	3.0500	H19B···C3^vi^	3.5619
C3···H1A^vi^	3.5331	H19B···C4^vi^	3.3424
C3···H14A^iv^	3.1507	H19B···H3^vi^	3.2350
C3···H18B^vi^	3.1087	H19B···H4^vi^	2.8224
C3···H19B^vi^	3.5619	H19B···H13A^i^	3.5436
C4···H10^iv^	3.5768	H19B···H13B^i^	2.9943
C4···H14A^iv^	3.5164	H19B···H19A^xiii^	3.0665
C4···H15B^iv^	3.5109	H19B···H20A^xiii^	3.0827
C4···H18B^vi^	3.3700	H20A···C4^x^	3.1710
C4···H19B^vi^	3.3424	H20A···C5^x^	3.4057
C4···H20A^xi^	3.1710	H20A···C13^i^	3.5946
C5···H9^iv^	3.536 (19)	H20A···C19^xiii^	3.3249
C5···H10^iv^	2.9791	H20A···H4^x^	2.5151
C5···H14B^vii^	3.1512	H20A···H5^x^	2.9903
C5···H20A^xi^	3.4057	H20A···H13A^i^	2.9036
C5···H20B^xi^	3.5141	H20A···H13B^i^	3.4126
C6···H1A^vi^	3.5387	H20A···H19A^xiii^	2.6815
C6···H9^iv^	3.181 (19)	H20A···H19B^xiii^	3.0827
C6···H10^iv^	3.5256	H20B···O2^ix^	2.6927
C6···H14B^vii^	3.2197	H20B···O4^ix^	3.1709
C6···H18A^iii^	3.2131	H20B···C5^x^	3.5141
C7···H1A^vi^	2.8961	H20B···H1B^viii^	3.3308
C7···H9^iv^	3.448 (19)	H20B···H4^x^	3.4074
C7···H15A^i^	3.1974	H20B···H4A^ix^	2.8906
C8···H1A^vi^	3.1454	H20B···H5^x^	3.0067
			
C1—O1—C2	118.76 (13)	H1B—C1—H1C	109.479
O1—C2—C3	123.88 (13)	C2—C3—H3	119.939
O1—C2—C7	115.66 (13)	C4—C3—H3	119.939
C3—C2—C7	120.46 (14)	C3—C4—H4	119.629
C2—C3—C4	120.12 (15)	C5—C4—H4	119.623
C3—C4—C5	120.75 (16)	C4—C5—H5	120.477
C4—C5—C6	119.06 (15)	C6—C5—H5	120.466
C5—C6—C7	122.10 (14)	C5—C6—H6	118.958
C2—C7—C6	117.51 (13)	C7—C6—H6	118.945
C2—C7—C8	119.33 (13)	C7—C8—H8	115.8 (9)
C6—C7—C8	123.08 (12)	C9—C8—H8	117.0 (9)
C7—C8—C9	127.10 (14)	C8—C9—H9	119.3 (10)
C8—C9—C10	125.51 (14)	C10—C9—H9	115.1 (10)
C9—C10—C11	112.43 (11)	C9—C10—H10	104.489
C9—C10—C22	113.82 (11)	C11—C10—H10	104.490
C11—C10—C22	115.61 (12)	C22—C10—H10	104.498
C10—C11—C12	122.53 (13)	C12—C13—H13A	109.122
C10—C11—C16	118.62 (13)	C12—C13—H13B	109.121
C12—C11—C16	118.75 (13)	C14—C13—H13A	109.120
O4—C12—C11	122.89 (13)	C14—C13—H13B	109.119
O4—C12—C13	114.90 (15)	H13A—C13—H13B	107.853
C11—C12—C13	122.20 (15)	C13—C14—H14A	109.586
C12—C13—C14	112.40 (15)	C13—C14—H14B	109.587
C13—C14—C15	110.32 (15)	C15—C14—H14A	109.592
C14—C15—C16	112.19 (15)	C15—C14—H14B	109.587
O5—C16—C11	122.86 (13)	H14A—C14—H14B	108.131
O5—C16—C15	116.05 (14)	C14—C15—H15A	109.166
C11—C16—C15	121.08 (15)	C14—C15—H15B	109.165
O2—C17—C18	116.30 (16)	C16—C15—H15A	109.164
O2—C17—C22	123.05 (15)	C16—C15—H15B	109.166
C18—C17—C22	120.64 (14)	H15A—C15—H15B	107.891
C17—C18—C19	111.76 (16)	C17—C18—H18A	109.268
C18—C19—C20	108.65 (14)	C17—C18—H18B	109.261
C19—C20—C21	112.65 (15)	C19—C18—H18A	109.270
O3—C21—C20	114.76 (14)	C19—C18—H18B	109.262
O3—C21—C22	122.93 (15)	H18A—C18—H18B	107.941
C20—C21—C22	122.31 (16)	C18—C19—H19A	109.957
C10—C22—C17	122.56 (13)	C18—C19—H19B	109.959
C10—C22—C21	119.72 (14)	C20—C19—H19A	109.965
C17—C22—C21	117.58 (14)	C20—C19—H19B	109.962
C21—O3—H3A	109.468	H19A—C19—H19B	108.341
C12—O4—H4A	109.461	C19—C20—H20A	109.067
O1—C1—H1A	109.469	C19—C20—H20B	109.070
O1—C1—H1B	109.475	C21—C20—H20A	109.054
O1—C1—H1C	109.465	C21—C20—H20B	109.061
H1A—C1—H1B	109.469	H20A—C20—H20B	107.825
H1A—C1—H1C	109.470		
			
C1—O1—C2—C3	0.5 (3)	C10—C11—C12—C13	172.78 (13)
C1—O1—C2—C7	−178.50 (14)	C10—C11—C16—O5	7.5 (3)
O1—C2—C3—C4	−178.93 (15)	C10—C11—C16—C15	−173.79 (12)
O1—C2—C7—C6	178.35 (13)	C12—C11—C16—O5	−169.16 (15)
O1—C2—C7—C8	1.5 (3)	C12—C11—C16—C15	9.5 (3)
C3—C2—C7—C6	−0.7 (3)	C16—C11—C12—O4	167.84 (15)
C3—C2—C7—C8	−177.51 (15)	C16—C11—C12—C13	−10.7 (3)
C7—C2—C3—C4	0.0 (3)	O4—C12—C13—C14	161.33 (14)
C2—C3—C4—C5	0.8 (3)	C11—C12—C13—C14	−20.0 (3)
C3—C4—C5—C6	−1.0 (4)	C12—C13—C14—C15	50.1 (2)
C4—C5—C6—C7	0.3 (3)	C13—C14—C15—C16	−51.0 (2)
C5—C6—C7—C2	0.5 (3)	C14—C15—C16—O5	−159.17 (16)
C5—C6—C7—C8	177.25 (16)	C14—C15—C16—C11	22.0 (3)
C2—C7—C8—C9	−162.38 (15)	O2—C17—C18—C19	−153.73 (12)
C6—C7—C8—C9	21.0 (3)	O2—C17—C22—C10	6.0 (2)
C7—C8—C9—C10	179.16 (14)	O2—C17—C22—C21	−169.71 (12)
C8—C9—C10—C11	153.67 (15)	C18—C17—C22—C10	−175.32 (12)
C8—C9—C10—C22	19.8 (3)	C18—C17—C22—C21	9.0 (2)
C9—C10—C11—C12	−43.38 (19)	C22—C17—C18—C19	27.47 (19)
C9—C10—C11—C16	140.09 (13)	C17—C18—C19—C20	−55.86 (18)
C9—C10—C22—C17	53.17 (17)	C18—C19—C20—C21	50.0 (2)
C9—C10—C22—C21	−131.23 (12)	C19—C20—C21—O3	164.22 (14)
C11—C10—C22—C17	−79.22 (15)	C19—C20—C21—C22	−15.3 (3)
C11—C10—C22—C21	96.37 (15)	O3—C21—C22—C10	−10.8 (2)
C22—C10—C11—C12	89.66 (16)	O3—C21—C22—C17	165.03 (13)
C22—C10—C11—C16	−86.88 (16)	C20—C21—C22—C10	168.73 (13)
C10—C11—C12—O4	−8.7 (3)	C20—C21—C22—C17	−15.5 (2)

Symmetry codes: (i) *x*−1/2, −*y*+1/2, *z*−1/2; (ii) −*x*+1, −*y*+1, −*z*; (iii) −*x*+1/2, *y*−1/2, −*z*+1/2; (iv) −*x*+1, −*y*, −*z*; (v) −*x*+3/2, *y*+1/2, −*z*+1/2; (vi) −*x*, −*y*, −*z*; (vii) −*x*+3/2, *y*−1/2, −*z*+1/2; (viii) *x*+1/2, −*y*+1/2, *z*+1/2; (ix) −*x*+1/2, *y*+1/2, −*z*+1/2; (x) *x*, *y*+1, *z*; (xi) *x*, *y*−1, *z*; (xii) *x*−1, *y*, *z*; (xiii) −*x*, −*y*+1, −*z*; (xiv) *x*+1, *y*, *z*.

### Hydrogen-bond geometry (Å, °)

**Table:**

*D*—H···*A*	*D*—H	H···*A*	*D*···*A*	*D*—H···*A*
O3—H3A···O5	0.82	1.85	2.644 (3)	163.
O4—H4A···O2	0.82	1.80	2.594 (3)	162.
C19—H19A···O4^ix^	0.97	2.49	3.272 (3)	137.

Symmetry codes: (ix) −*x*+1/2, *y*+1/2, −*z*+1/2.
